# Epidemiology of COVID-19 among Children and Adolescents in Sudan 2020–2021

**DOI:** 10.3390/epidemiologia4030025

**Published:** 2023-06-23

**Authors:** Amna Khairy, Narmin Elhussein, Omer Elbadri, Sanad Mohamed, Elfatih M. Malik

**Affiliations:** 1 Sudan FETP Graduates, Federal Ministry of Health, Khartoum 11111, Sudan; 2 FETP Technical Coordinator, EMPHNET, Khartoum 11111, Sudan; 3 Sudan FETP Graduates, Blue Nile National Institute for Communicable Disease, Gezira 21111, Sudan; 4 Associate Professor of Community Medicine, Department of Community Medicine, Faculty of Medicine, University of Khartoum and GHD/EMPHNET Consultant for EBS in Sudan, Khartoum 11111, Sudan

**Keywords:** COVID-19, surveillance, epidemiology, reported incidence, odds ratios, children, adolescents, Sudan

## Abstract

Children and adolescents account for a small proportion of confirmed COVID-19 cases, with mild and self-limiting clinical manifestations. The distribution and determinants of COVID-19 among this group in Sudan are unclear. This study used national COVID-19 surveillance data to study the epidemiology of COVID-19 among children and adolescents in Sudan during 2020–2021. A cross-sectional study was performed to estimate the reported incidence of children and adolescents with COVID-19; the clinical features; and the mortality among those who tested positive for COVID-19. A total of 3150 suspected cases of COVID-19 infection fulfilled the study criteria. The majority of cases were above 10 years of age, 52% (1635) were males, and 56% (1765) were asymptomatic. The reported incidence rates of COVID-19 among children and adolescents in Sudan was 1.3 per 10,000 in 2021. Fever, cough, and headache were the most frequent symptoms reported among the suspected cases. The case fatality rate was 0.2%. Binary logistic regression revealed that loss of smell was the most significantly associated symptom with a positive test. We recommend further study to identify risk factors. Additionally, we recommend including these age groups in the vaccination strategy in Sudan.

## 1. Introduction

Following the identification of clusters of pneumonia cases in Wuhan, China in December 2019 and the WHO’s declaration of the coronavirus disease (COVID-19) pandemic on 12 March 2020 [1,2], 760,360,956 cases and 6,873,477 deaths were reported globally up to March 2023. The Eastern Mediterranean and Afro regions were the most affected WHO regions. Despite having the lowest number of cases reported, with 23,276,221 and 9,509,465 cases reported, respectively, the highest case fatality occurred in these two regions; 349,752 and 175,313 deaths were reported, with 1.5% and 1.8% case fatality rates in the EMRO and Afro regions, respectively, compared to the global case fatality rate of 0.9%.

Overall, the majority of cases are adults, and children account for a small proportion of the reported confirmed cases; more reported cases are seen among 5-to-14-year-old children in comparison to children under 5, which represented 6.3% (6,020,084) and 1.8% (1,695,265) of global cases, respectively, during 2019–2021 [3]. However, this could be underestimated due to their tendency to have asymptomatic infection [4,5]. The latter has been variably reported to range from 10% to 90% of pediatric COVID-19 cases [6], and different host-related theories have been postulated as physio-pathological explanations [7].

Generally, the clinical course of COVID-19 among children is relatively mild, with a wide range of symptoms, including: fever, cough, diarrhea, vomiting, headache, and loss of appetite [8,9]. However, susceptibility to severe clinical outcomes varies according to many factors, such as virus characteristics, presence of co-infection(s), and the underlying health condition of the child. Tendency to develop severe disease was variably reported across different genetic variants of the virus, with disease caused by the alpha variant having a more severe clinical course among children compared to infections of Omicron [10]. In addition, a severe clinical course of multi-system inflammatory syndrome has been characteristically reported among children with COVID-19 infection. This condition was associated with the need for intensive care and mortality in 63% and 10% of patients, respectively [11,12]. Furthermore, even with a mild clinical course, children may suffer long-term symptoms following the infection, significantly affecting their quality of life; a condition described by the WHO using the term “post COVID-19 condition”, and generally known as “long COVID” [13]. Long-term post-discharge psychological and mental health outcomes have been reported among children previously admitted with COVID-19 infection [14]; high risk for anxiety and depression was found among children even with mild COVID-19 infection [15]; and developmental manifestations have also been reported [13,16].

Family contact is a major route of transmission for COVID-19 infection to children, but it is unclear whether children play a significant role in transmitting the infection among them and/or to adults [17]. Some evidence has shown that children may be less infectious, as measured by secondary attack rates, than adolescents and adults [18], especially in the context of overall low community transmission [3]. Nevertheless, outbreaks have been reported from secondary schools, summer camps, and day care centers, especially when neither physical distancing nor masks were used to reduce the risk of transmission [19,20]; several studies have also reported that adults have a higher risk for infection from a child compared to an adult within the same household [20]. Moreover, despite poor evidence on the mechanism of intrauterine fetal transmission, newborn infants have developed symptomatic COVID-19 [8].

Up to March 2023 in Sudan, 63,853 COVID-19 cases and 5023 deaths were reported, with a case fatality rate of 7.9%. The elderly, health care workers, and adults were considered as high-risk groups for infection, and were the first population groups prioritized to receive the COVID-19 vaccination accordingly [21,22]. High-income countries have already prioritized children to receive the vaccination. This is a key COVID-19 control measure and helps to prevent severe outcomes. However, in the context of Sudan and other low-income countries, this decision might be hindered by the scarcity of evidence on the burden and clinical characterization of the disease among this group [23]. This study aims to describe the epidemiology of COVID-19 in children and adolescents in Sudan during 2020–2021.

## 2. Materials and Methods

### 2.1. Study Design Setting and Participants

A cross-sectional study using national COVID-19 surveillance data was conducted. The suspected COVID-19 cases were reported from all 18 states.

### 2.2. Sample Size and Sampling Considerations

The study includes all children and adolescents below the age of 19 years who had SARS-CoV-2 PCR test results from nasopharyngeal swab during the period from March 2020 to December 2021. Age group categorization was based on the WHO’s age classification as follows: less than 1 year (post-neonate/infant), from 1 to 4 years (young children), 5 to 9 years (older children), 10 to 14 years (young adolescents), and 15 to 19 years (older adolescents) [24].

### 2.3. Study Variables and Data Collection

The study variables were extracted from the national COVID-19 surveillance Line list. Patients’ socio-demographics, testing status, clinical feature, and outcomes were obtained for each eligible case using an extraction form.

### 2.4. Data Management and Statistical Analysis

Data cleaning was performed by removing duplicates. Data analysis was conducted using SPSS version 23. A descriptive analysis in the form of frequencies and percentages was conducted. The reported incidence of COVID-19 infection among children and adolescents was calculated; the nominator was those who tested positive for SARS-CoV-2 infection, and the denominator was the population of 1,937,482 which represented the total number of children and adolescents at risk for COVID-19 infection, as per the estimated projected population by the Sudan central Bureau of statistics 2021. Bivariate analysis was conducted using the Chi-squared test to study the factors associated with positive COVID-19 tests among children and adolescents, and multivariable logistic regression analysis was conducted at 95% confidence level and *p* < 0.05. The outcome variable was COVID-19 test results measured as dichotomous variable, negative or positive, and independent variables were categorical variables. These include: age (<1 year, 1–4 years, 5–9 years, 10–14 years, 15 and more), gender (male/female) and clinical presentation (symptomatic/asymptomatic). In addition, presenting symptoms were defined as: fever (Yes/No), cough (Yes/No), shortness of breath (Yes/No), sore throat (Yes/No), headache (Yes/No), loss of smell and taste (Yes/No), joint pain (Yes/No), muscle pain (Yes/No), back pain (Yes/No), fatigue (Yes/No), vomiting (Yes/No), diarrhea (Yes/No), nausea (Yes/No), loss of consciousness (Yes/No), runny nose (Yes/No), and loss of appetite (Yes/No).

## 3. Results

### 3.1. Demographic and Clinical Characteristics of Study Participants

A total of 3150 suspected cases for COVID-19 infection fulfilled the inclusion criteria of the study. The majority (85.9%) of cases were above 10 years of age and 67.0% (2112) of the cases were among the age group of 15 years and above, while around 6% (188) of the cases were under 5 years of age. Almost 52% (1635) of the cases were males, and 56.03% (1765) of the cases were asymptomatic, as shown in Table 1.

### 3.2. Descriptive Statistics

**Figure 1:**
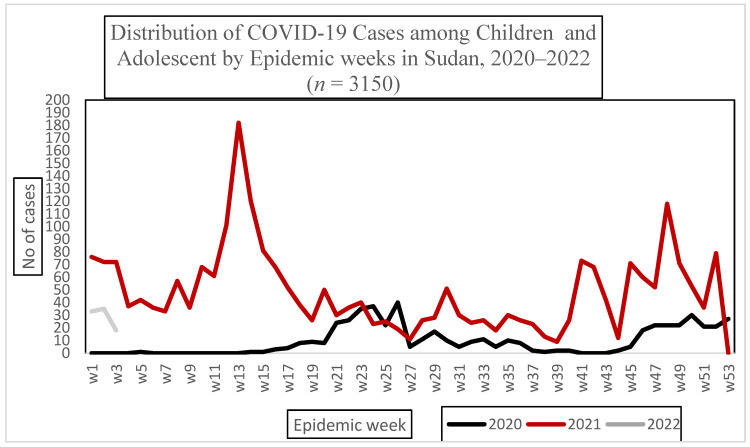
Incidence of confirmed COVID-19 infection among children and adolescents per 10,000 population per state—Sudan 2020–2021.

The reported incidence rate of COVID-19 among children and adolescents in 2021 in Sudan was 1.3 per 10,000 population per year, Figure 1. The high incidence of COVID-19 infection in Sudan was seen in states cross bordering other countries. Less than half of the cases (43%) tested positive. Fever, cough, headache, and sore throat were reported among 22% (866), 18% (735), 16% (658) and 14% (581) of the cases, respectively (Table 1).

### 3.3. Factors Associated with COVID-19 Infection among Children and Adolescents in Sudan 2021

Age was positively associated with laboratory-confirmedCOVID-19 infection. A total of 948 (45%) of cases suspected to have COVID-19 infection within the age group 15 years and above, tested positive for SARS-CoV-2 virus. In comparison, only 41% (247) and 36% (101) of the age group 10–14 years and 5–9 years tested positive, respectively (Table 2). Adjusted odds ratios (OR) showed that, this association was statistically significant; OR (95% CI) was 3.8 (1.9–6.4), 3.7 (2.0–6.9), 3.1 (1.6–5.9) and 3.0 (1.4–6.3) for the age groups 15 years and above, 10–14 years, 5–9 years and 1–4 years, respectively (Table 3). Females suspected to have COVID-19 infection were more likely to test positive compared to males; OR (95% CI) 1.3 (1.1–1.5). Among all the reported symptoms from suspected cases for COVID-19 infection, loss of taste and smell was the only statistically significant symptom associated with positive SARS-CoV-2 PCR test; OR (95% CI): 2.1 (1.4–3.3). By Contrast, headache and runny nose were inversely associated with positive SARS-CoV-2; with an OR (95% CI) of 0.7, (0.5–0.9) and 0.6 (0.3–0.9).

## 4. Discussion

Overall, the reported incidence rate of COVID-19 among children and adolescents in Sudan 2021 was 1.3 per 10,000 population, which was lower than in adults (9.2 per 10,000 population). This matches the results from a case series of all 1212 confirmed cases in Henan province, China, from 24 January 2020 to 14 February 2020, which reported a similarly low proportion of affected children [25].

Adolescent age groups (10–19) years were more likely to test positive for COVID-19 infections compared to younger children. This agrees with what was reported in a study conducted in Bauchi State, North-East Nigeria, where 81.1% of the patients were adolescents and fell within the age group of 15–19 years [26] and had seroprevalence comparable to adults in some studies [27]. However, this age group has not been prioritized yet to receive COVID-19 vaccination; according to the national COVID-19 vaccination policy in Sudan, only adults (above 18 years old) are eligible for COVID-19 vaccination.

In this study, female children and adolescents suspected to have COVID-19 infection were 30% more likely to test positive. There is inconsistency in the evidence on gender difference of COVID-19 infection among children and adolescents globally, Nyimas et al. reported male children were at higher risk of being infected with COVID-19 than females in Jakarta, Indonesia [28]. However, other systematic review reported no gender difference for the infections among this group [29]. Additionally, studies have reported no gender difference in COVID-19 infection among adults; however, adult males were reported to have worsening, severe COVID-19 infection compared to females [30]. The results revealed a low case fatality rate among children and adolescents (0.2%), and all mortalities were among non-laboratory-confirmed COVID-19 infections. This finding is consistent with the global trend, where the majority of cases had mild-to-moderate clinical features. Only 0.6% of these developed severe disease, and 0.3% developed critical manifestations of disease [3].

In this study, the only statistically significant clinical predictor for COVID-19 among children and adolescents was the loss of smell. This finding is consistently reported among both children and adults. While it may take a transient course [31], it is also associated with the development of long COVID in both children and adults [13,32].

In contrast, children presenting with headache were less likely to test positive; this could be linked to the non-specific nature and subjectivity of headache as a presenting symptom. Other commonly presenting symptoms in suspected cases were fever (22%), cough (18%), and sore throat (14%); these findings were consistent with a meta-analysis study that described the clinical manifestation of COVID-19 in children as the following: cough (49%), fever (47%), and sore throat (36%) [29]. However, these symptoms had no statistically significant association with positive COVID-19 tests.

### Limitations of the Study

Due to the lack of molecular characterization of COVID-19 infections in Sudan, the epidemiology of COVID-19 among children in this study could not be compared against different viral variants.

In addition, only secondary data from national COVID-19 surveillance was used, limiting the study of other clinical characteristics of COVID-19 among children, which are not routinely included as part of surveillance, including psychological, mental, and development symptoms.

As no retrospective follow up was performed, this study did not address long COVID or post-COVID conditions, a problem that has been linked to the loss of smell, which was identified in this study as one of the predictors for COVID-19 among children.

## 5. Conclusions

Overall, the incidence of COVID-19 among children was lower than that of adults, but adolescents showed higher risk for the disease compared to young children. Hence, the eligibility of this age group needs to be revised for potentially receiving COVID-19 vaccination as part of the national COVID-19 vaccination policy in Sudan, and perhaps other comparable contexts in low-income countries. The study’s findings on the symptomatology of COVID-19 among children can be used to support the development of a clinical diagnostic algorithm for COVID-19 among children and adolescents. Loss of smell can be used as rule of thumb for triaging in low-resource settings with low COVID-19 testing capacities.

## Figures and Tables

**Table 1:** Socio-demographic and clinical characteristics of study participants children and adolescents (less than 19 years) suspected with COVID-19 infection—Sudan 2020-2021 (*n* = 3150).

Background Characteristics	*n*	(%)	
Age	<1 year	79	2.5
	1–4 years	88	2.8
	5–9 years	278	8.8
	10–14 years	593	18.8
	15 and over	2112	67.1
Gender	Male	1635	51.9
	Female	1515	48.1
Result of PCR	Negative	1809	57.4
	Positive	1341	42.6
Present of symptoms	Symptomatic	1385	44.0
	Asymptomatic	1765	56.0
* Clinical Presentation			
	Fever	866	27.5
	Cough	735	23.3
	Shortness of breath	393	12.5
	Sore throat	581	18.4
	Headache	658	20.9
	Loss of smell and taste	159	5.0
	joint pain	100	3.2
	Muscle pain	106	3.4
	Back pain	16	0.5
	Fatigue	130	4.1
	Vomiting	14	0.4
	Diarrheal	66	2.1
	Nausea	08	0.3
	Unconsciousness	04	0.1
	Runny Nose	104	3.3
	loss of appetite	05	0.2

* as each case may have more than one presenting symptoms, symptoms adds up to more than N (3150).

**Table 2:** Cross-tabulation and Pearson Chi-square test for factors associated with positive COVID-19 test among children and adolescents in Sudan 2020–2021.

Factors Associated with Positive COVID-19 Test		Result		Chi-Squared	*p*-Value
		Negative	Positive		
Age	<1 year	66 (83.5%)	13 (16.5%)	32.688	0.001
	1–4 years	56 (63.6%)	32 (36.4%		
	5–9 years	177 (63.6%)	101 (36.4%)		
	10–14 years	346 (58.3%)	247 (41.7%)		
	15 and more	1164 (55.1%)	948 (44.9%)		
Gender	Male	951 (58.4%)	677 (41.6%)	1.886	0.170
	Female	844 (56%)	664 (44.0%)		
Symptoms	Asymptomatic	905 (51.2%)	860 (48.8%)	62.179	0.001
	Fever (yes)	548 (63.2%)	318 (36.8%	16.724	0.001
	Cough (yes)	495 (67.3%)	240 (32.7%)	38.576	0.001
	Shortness of breath (yes)	261 (66.4%)	132 (33.6%)	14.823	0.001
	Sore throat (yes)	371 (63.8%)	210 (36.2%)	12.036	0.01
	Headache (yes)	441 (67%)	217 (33%)	31.306	0.001
	Loss smell and taste (yes)	72 (45.3%)	87 (54.7%)	10.104	0.001
	Joint pain (yes)	69 (69%)	31 (31%)	5.656	0.017
	Muscle pain (yes)	78 (73.5%)	28 (26.5%)	11.711	0.001
	Back pain (yes)	12 (75%)	04 (25.0%)	2.031	0.15
	Fatigue (yes)	79 (60.8%)	51 (39.2%)	0.619	0.431
	Vomiting (yes)	11 (78.6%)	03 (21.4%)	2.571	0.109
	Diarrhea (yes)	44 (66.7%)	22 (33.3%)	2.353	0.125
	Nausea (yes)	05 (62.5%)	03 (37.5%)	0.084	0.771
	Unconsciousness (yes)	02 (50%)	02 (50%)	0.090	0.764
	Runny Nose (yes)	80 (76.9%)	24 (23.1%)	16.718	0.001
	Loss of appetite (yes)	03 (60%)	02 (40%)	0.014	0.907
Outcome	Death	06 (100%)	0 (0%)	8.109	0.004

**Table 3:** Adjusted OR, 95% CI for factors associated with positive COVID-19 test among children and adolescents in Sudan 2021—multivariable logistic regression analysis.

**Factors Associated with Positive COVID-19 Test**		**OR**	**95% C.I**	**P**
Age	<1 year			
	1–4 years	3.0	(1.4–6.3)	0.001
	5–9 years	3.1	(1.6–5.9)	0.001
	10–14 years	3.7	(2.0–6.9)	0.001
	15 and over	3.4	(1.9–6.4)	0.001
Gender	Female	1.3	(1.1–1.5)	0.01
	Male			
Symptoms	Fever	1.0	(0.7–1.2)	0.72
	Cough	0.8	(0.6–1.0)	0.04
	Shortness of breath	1.0	(0.7–1.3)	0.78
	Sore throat	1.0	(0.8–1.3)	0.91
	Headache	0.7	(0.5–0.9)	0.001
	Loss of smell and taste	2.1	(1.4–3.3)	0.001
	Joint pain	1.4	(0.7–2.9)	0.31
	Muscle pain	0.7	(0.3–1.3)	0.25
	Back pain	0.5	(0.1–1.7)	0.28
	Fatigue	1.3	(0.8–2.0)	0.32
	Vomiting	0.5	(0.1–1.9)	0.31
	Diarrhea	0.9	(0.5–1.7)	0.79
	Nausea	1.9	(0.4–9.6)	0.42
	Unconsciousness	1.3	(0.2–9.6)	0.77
	Runny Nose	0.6	(0.3–0.9)	0.02
	Loss of appetite	1.3	(0.2–8.6)	0.82

## Data Availability

The data that support the findings of this study are available from the Sudan national surveillance system.
